# Quantitative Photo Activated Localization Microscopy: Unraveling the Effects of Photoblinking

**DOI:** 10.1371/journal.pone.0022678

**Published:** 2011-07-26

**Authors:** Paolo Annibale, Stefano Vanni, Marco Scarselli, Ursula Rothlisberger, Aleksandra Radenovic

**Affiliations:** 1 Laboratory of Nanoscale Biology, Institute of Bioengineering, Ecole Polytechnique Fédérale de Lausanne, Lausanne, Switzerland; 2 Laboratory of Computational Chemistry and Biochemistry, Institute of Chemical Sciences and Engineering, Ecole Polytechnique Fédérale de Lausanne, Lausanne, Switzerland; Friedrich-Schiller-University Jena, Germany

## Abstract

In this work we discuss how to use photophysical information for improved quantitative measurements using Photo Activated Localization Microscopy (PALM) imaging. We introduce a method that reliably estimates the number of photoblinking molecules present in a biological sample and gives a robust way to quantify proteins at the single-cell level from PALM images. We apply this method to determine the amount of β2 adrenergic receptor, a prototypical G Protein Coupled Receptor, expressed on the plasma membrane of HeLa cells.

## Introduction

Super-resolution techniques based on the sequential photoswitching/photo-activation [1], [2], [3], [4], [5], [6], [7], [8], [9] of single photo-emitters have allowed detection of single molecules with spatial localization accuracy below 10 nanometers and Nyquist-Shannon-limited resolution [10] of approximately 20 nm. In the case of PALM, the key idea is that super-resolution images are constructed from rounds of photo-activating sparse subsets of a sample, allowing the localization of those single emitters with high precision, building up over time the complete image with high spatial resolution.

Although several groups have reported super-resolution images of cellular structures in living or fixed cells [7], [8], [9], [11], [12], [13], so far only a few studies used PALM/STORM data in a quantitative way [14] [15] [16]. Advanced fluorescence microscopy in general has facilitated quantitative investigation of protein stoichiometry and dynamics under physiological conditions. It was for example used to measure global and local concentrations of 28 cytoskeletal and signaling proteins in yeasts [17], stochastic nature of gene expression in bacteria [18] and low copy number membrane receptors in insect cells [19]. All these and similar studies would benefit greatly from the increased spatial resolution afforded by PALM. Why are quantitative PALM reports still rare? Besides the novelty of the technique and the demanding experimental setup, the lack of quantitative PALM experiments is most likely due to our limited knowledge of fluorophore photophysics. For an ideal photoactivatable fluorescent protein one would observe, after photoactivation, continuous emission and photobleaching, allowing for a PALM measurement to become intrinsically a counting experiment. Departures of different magnitude from this condition, depending on the fluorophore chosen, may occur [20], [21], [22], [23], [24], [25], [26], [27], [28], [29], requiring a strategy to infer the original number of molecules beneath their photophysical behavior. In particular, the fluorescent proteins used in PALM may display triplet blinking, reported to be in the millisecond time range and therefore most often filtered out by a detector integration times larger than 10 ms. In addition to this, the presence of dark states from which the molecule can spontaneously recover, or blink, on timescales ranging from hundreds of milliseconds to many seconds is another common fluorophore feature. Dating back to 1997 the presence of a long-lived dark state recovered by near-UV irradiation was discovered for certain GFP mutants [30].

Here, we investigate the impact of fluorophore photoblinking on a PALM experiments in order to devise an effective strategy to determine accurate protein concentrations and obtain spatial point patterns devoid of photoblinking-induced artifacts.

## Results

**Figure 1:**
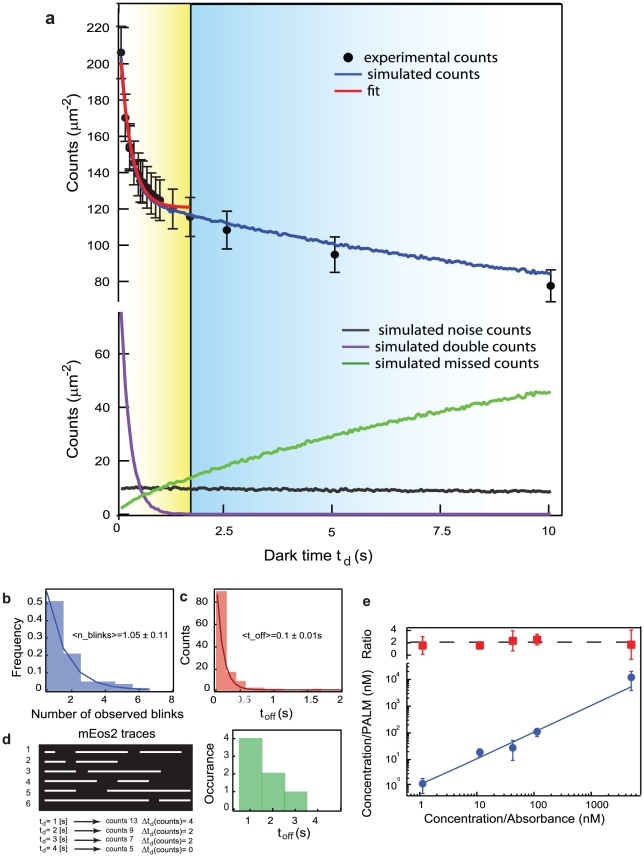
Protein counting in vitro and in silico. **a**) Experimental (markers) and simulated (blue line) total numbers of mEos2 molecules localized as a function of dark time *t_d_*, where dark time *t_d_* is time allowed for a molecule to go dark before being identified as a different molecule when fluorescence resumes. Simulated counts, out of total, ascribed respectively to missed counts (green), multiple counts (pink) and noise (gray). For all samples the duration of the acquisition is 20000×50 ms frames. Red curve shows best fit to data for dark time *t_d_* values comprised between 0.05 s and 2 s. If no missed counts were to occur, the asymptote of the decaying curve of the observed counts would converge to the effective number of molecules present in the sample. Fitting to the equation (1) yields *t_off_* = 0.2±0.1 s and <*n_blinks_*> = 0.7±0.1 consistent with what reported respectively in b) and c) and **Figure S2.** *N* from the fit yields *N* = 121±6 molecules/µm^2^ whereas the total density of the simulated sample is 135 molecules/µm^2^ including noise counts, giving a 10% agreement. **b**) Histogram of the number of times an mEos2 molecule undergoes photoblinking before definitive photobleaching. Experimental values and single exponential best fits are shown, 1/e decay values is <*n__blinks_*> = 1.05±0.11. **c**) Histogram of the measured off-times *t_off-high_* = 0.1±0.01 s **d**) Left: single molecule kymographs of 6 blinking/reactivating molecules. The duration of the dark times spans from 1 to 4 units (0.05–0.15 s), resulting in the off-times histograms on the right. If now molecular localizations are performed considering a dark time *t_d_* as 0.05 s, 0.1 s and 0.15 s the resulting number of counts are 13, 9, and 7 respectively. The difference between these values corresponds to the values of the off-time histogram **e**) Calibration curve for the concentrations extracted by fitting eq. 1 to PALM data. Blue circles: reconstructed concentration from PALM experiment (0.06 molecules/µm^2^/nM in TIRF) vs absorbance-derived concentration and best fit. Each point corresponds to the average of PALM experiments on three different samples. Red markers display the ratio between the concentration extracted directly from PALM at *t_d_* = 0.05 s and the concentration obtained using our method.

The correct identification of single molecules in dense samples of photoactivatable fluorescent proteins is strongly affected by molecular photoblinking. The number of times that a molecule can go off and then reappear in its bright state as well as the distribution of the fluorescence off-times are the crucial parameters that have to be taken into account in order to determine a threshold either on the fluorophore reactivations or on the duration of its dark state. After collecting by PALM imaging a large number of fluorescence traces separated by dark intervals (**Figure S1**), it is necessary to attribute them to specific molecules. Markers in **Figure 1** **a** display how grouping fluorescent traces in time according to a varying allowed dark time threshold *t_d_* results in an exponentially decreasing number of localized molecules. For a very short dark time *t_d_* (*t_d_*  = 0.05 s, corresponding to 1 camera frame) two bursts of fluorescence in the same spot separated only by a single dark frame are localized as two different molecules. For longer dark time *t_d_* values, blinking molecules are allowed to spend longer time in their dark state before being identified as different molecules and so the number of molecular counts decreases as shown in **Figure 1d** **.**

Depending on the fluorophore photophysics, excitation light power and acquisition frame rate, the choice of a certain dark time *t_d_* during data analysis and fluorescent trace grouping will result in a number of molecular localizations that might be far from the actual number of labeled molecules. If the average off-time of the blinking fluorophore exceeds the frame rate of the acquisition, then a dark time *t_d_* = 0.05 s will clearly result in significant overcounting. On the other hand, a longer *t_d_* might avoid counting the same molecule twice, but in a densely labeled sample it might induce grouping of fluorescent traces belonging to neighboring but different molecules (i.e. activated within the same detector's pixel). From (**Figure 1** **a**) it is clear that the number of localized counts is dramatically influenced by the choice of a dark time *t_d_* and that it is connected to the number of photoblinks a molecule can undergo. This observable is elusive to direct measurement due to the stochastic nature of both photoactivation and photoblinking. Therefore, to gain a deeper understanding of how the single molecule behavior gives rise to such an ensemble curve and how it is related to the actual number of molecules in the sample we have devised an *in-silico* strategy to reproduce the outcomes of a PALM experiment by simulating the stochastic on/off dynamics of the photoactivatable fluorescent protein that undergoes photoblinking.

Starting from the observation that the distribution of the off-times from fluorescent traces of isolated single mEos2 molecules in PAGE can be approximated by a single exponential (**Figure S2)** with a time constant dependent on the value of the photoactivation power, we investigate the effects on a PALM experiment of a probe displaying a fluorescent dynamics based on the following states: inactivated, activated, dark, bleached. The inactivated to activated transition is irreversible, and in the case of mEos2 corresponds to the green to red photoconversion. The activated to dark transition is reversible. A discussion on the implications of using a model with two dark states, namely inactivated, activated, dark_1_, dark_2_ and bleached on our method can be found in **Text S1**.

We model the fluorescence dynamics of the photoactivated form of mEos2[31], [32] by introducing a dark state from which the molecule can be recovered upon irradiation with 405 nm light. The state of each fluorophore can either switch from activated to dark according to a stochastic time-discrete dynamics or irreversibly photobleach in such a way as to reproduce the measured distribution of on-times, off-times and number of photoblinks extracted from the analysis of the fluorescent traces of a sparse sample of protein embedded in a PAGE gel. Representative histograms are displayed in (**Figure 1** **b and c)** as well as in (**Figure S2**) (for details on the sample preparation see **Text S1**). The on/off dynamics of many fluorophores randomly distributed in space is then used to reconstruct a spatial localization pattern mimicking the typical conditions of a PALM experiment (details for the simulations are provided in **Text S1**). The resulting simulated counts displayed in (**Figure 1** **a)** allow us to clearly determine that the number of counts is characterized by two main regimes: for low dark time *t_d_* values, it is characterized by an overcounting modulated by the multiple counts of the same blinking fluorophore (purple curve). In the second regime, at higher dark time *t_d_* values, the number of missed counts becomes non-negligible (green curve). The simulated curve pinpoints that the real number of molecules is recovered only for a value of dark time *t_d_* where missed counts are balanced by double counts.

On one hand, once the fluorescent dynamics of an individual molecule is known, an ensemble experiment can be simulated to find the molecular density that best matches the measured curves (**Figure S3**). On the other hand, the dynamics of each fluorophore is dependent on the excitation and photoactivation parameters, in addition to its nanoscale environment. These two conditions determine variability from experiment to experiment in the distribution of the on-times, off-times and average number of photoblinks before photobleaching.

For this reason we should note here that the counts vs. dark time *t_d_* curve already contains a part of this photophysical information. As graphically summarized in **Figure 1** **d** the off-times constant can be directly derived from the counts vs. dark time *t_d_* curve, from the observation that off-times histogram bins correspond to differences in counts at subsequent dark time *t_d_* as long as the probability of activating two distinct fluorophores in the same pixel within *t_d_* is negligible. Furthermore, at dark time *t_d_*  = 0.05 s, the number of localized molecules corresponds to the total number of detected fluorescent bursts. In other words, it corresponds to the total number of labeled molecules in the sample added to the product of the total number of molecules in the sample and the average number of blinks per molecule. Therefore, if no missed counts were to occur, the asymptote of the decaying curve of the observed counts would converge to the effective number of molecules present in the sample.

**Figure 2:**
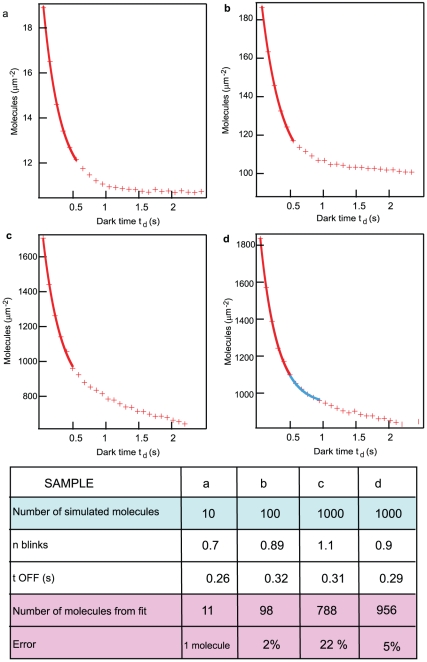
Semi-empirical fit to simulated data. Simulated PALM experiments on in-silico samples generated at three different densities while keeping the photophysical parameters constant. **a**) 10, **b**) 100 and **c**) and **d**) 1000 molecules/µm^2^ . The simulated counts curves are fitted to equation (1), using only the first 5 points of the curve, up to a *t_d_* = 50*10 ms = 0.5 s. **a**) 10 molecules/µm^2^ we observe an error of 1 particle/µm^2^ between the value extracted from the fit and the number of molecules present in the in-silico experiment, **b**) 100 molecules/µm^2^ we observe an error of 2% that goes up to **c**) 20% at 1000/µm^2^. However, upon reducing the photoactivation power in the densest sample **d**) this error reduces to approximately 5%. By reducing the photoactivation rate of a factor three, the fit can be extended (blue curve) up to 30 photoactivation cycles obtaining an error of 7%.

These considerations imply that the number of photoblinking fluorescent molecules *N* in the sample can be estimated from the number of counts at different dark time *t_d_*, *N*(*t_d_*), by fitting to the following semi-empirical equation:
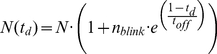
(1)in the regime of low dark time *t_d_* values. This approach is tested (**Figure 1e** **)** by plotting the number of calculated molecules (*N*) from PALM experiments against the concentration measured from absorbance in solution. The correlation observed from the fit is excellent over a broad range of concentrations, providing a clear validation of the method. To demonstrate the advantage of our method, we calculated the ratio (red markers **Figure 1** **e**) between the number of PALM counts at a fixed *t_d_* (e.g. *t_d_*  = 0.05 s) and number of molecules obtained using equation (1). From the obtained ratio it is obvious that our method prevents overcounting at all molecular densities. Our method of fitting the curve in the overcounting regime (small *t_d_* values) provides a reliable estimation of the number of molecules as far the photoactivation rate is kept reasonably low when the sample is dense. Although the fitting equation does not explicitly take into account missed counts the method appears to work in samples as dense as 1000 molecules/µm^2^ giving a result within at most 10% of the number of active molecules, as established by simulations and displayed in **Figure 2**.

**Figure 2** **a–c** displays the simulated counts vs *t_d_* curves for homogeneous samples generated with densities respectively of 10, 100 and 1000 molecules/µm^2^ while keeping the photophysical parameters fixed. Fitting is performed using only the first five points of the curve, in order to avoid the missed counts region as much as possible. The number of molecules extracted from the fit is in excellent agreement with the simulated number of molecules up to 100 molecules/µm^2^ declining to a 20% error only for a density of 1000 molecules/µm^2^ or above. However, upon the reduction of the photoactivation power (and accordingly of the missed counts) the fit yields a value with the much smaller error of 5%, as reported in the table.

**Figure 3:**
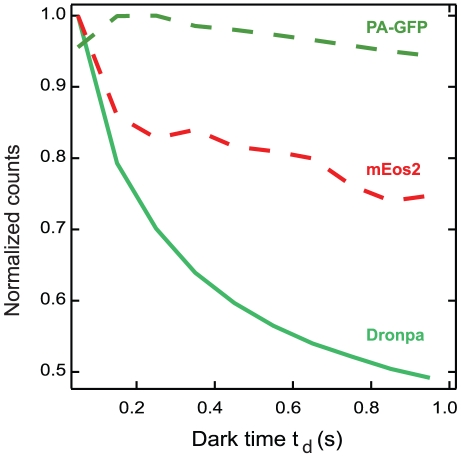
Comparison of counts vs *t_d_* curve for three photoactivatable fluorescent proteins. Normalized number of localized molecules vs *t_d_* for three different photo-activatable fluorescent proteins, PA-GFP, Dronpa and mEos2.

The photophysical dependence of the counts curve can also be found for other photoactivatable fluorescent proteins that can be used in a PALM experiment. We report in **Figure 3** three representative normalized counts curves for mEos2, PA-GFP [33] and Dronpa [34], [35]. Dronpa, a reversible photo-switcher, reaches an asymptote at a counts value that is approximately 50% of the initial counts, indication of a significant fraction of the sample being counted multiple times. PA-GFP displays a milder decay, an indication either of a reduced blinking or of the difficulty of detecting shorter bursts of fluorescence from this fluorophore. The latter explanation would be consistent with the rise of the curve at low *t_d_,* most likely due to the fact that the photon threshold for detection is achieved only collating a few successive bursts of fluorescence from the emitter. As detailed in **Text S1**, we have also extensively tested our method on in-vitro control experiments for varying intensities of the photoactivation source and for varying molecular densities (**Figures S4 and S5**). We generally observe a good agreement between measured photophysics at the single molecule level and values extracted from the fit to the PALM counts curves.

**Figure 4:**
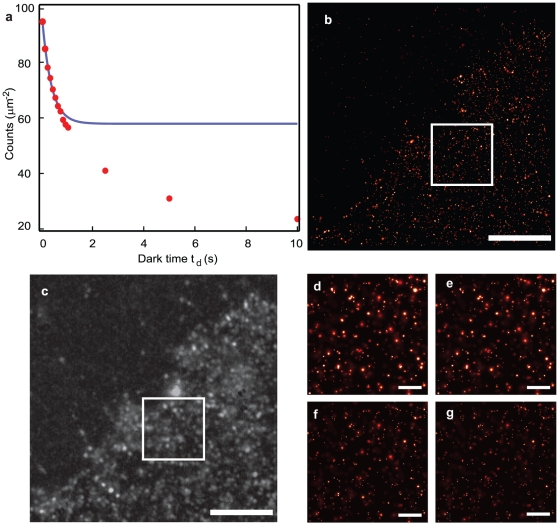
Estimation of molecular density of β2-adrenergic receptor. **a**) Red Markers - number of localized molecules as a function of dark time *t_d_* with density estimation in the cell. Molecules localized in a 1 µm^2^ region of the plasma membrane of the fixed HeLa cell expressing β2-mEos2 displayed in **b**). Blue line -fit using equation (1) limited to the overcounting regime yields an estimated density of approximately 60 molecules/µm^2^, t_off_ = 0.36±0.05 s and n_blink_ = 0.63±0.07. **b**) TIRF-PALM image of a region of the cell expressing β2-mEos2 (*t_d_* = 10 s, localization precision<35 nm) in **c**) its corresponding TIRF image **d**), **e**), **f**), and **g**) insets from a) displaying isolated localized receptors by a gaussian function for 4 different dark time values **d**) t_d_ = 0.5 s **e**) t_d_ = 1 s **f**) t_d_ = 5 s and **g**) t_d_ = 10 s.

We use this general framework on a real-life application of PALM experiments on the plasma membrane of fixed HeLa cells by determining the number of the labeled adrenergic receptor β2-mEos2. Although measurements of low copy number expression of this receptor have been previously reported [19], our method presents the advantage of allowing simultaneously imaging and concentration estimation over a wide expression range. The data reported in **Figure 4** **a** confirms that in our experiments in cells the counts vs. dark time *t_d_* curve retains an exponential decay, validating that the distribution of off-times is also exponential, at least at the ensemble level. The measured t_off_ falls close to the upper limit of the range 0.13 s to 0.37 s observed in in-vitro samples (**Figure S2**) and the average number of photoblinks is only slightly underestimated with respect to what is reported in **Figure 1** **.** The measured density in the sample we show in **Figure 4** is approximately 60 molecules/µm^2^. Our approach allows to accurately quantitative the number of labeled proteins even in relatively high expression systems, where the identification and subsequent counting of single molecules by conventional fluorescence microscopy is not possible anymore, as clearly displayed by the comparison of TIRF vs TIRF-PALM images (**Figure 4b** vs **Figure 4c**).

It should be noted that since the exponential behavior of the distribution reflects an ensemble property, it does not rule out a-priori that a small fraction of the molecules, whose contribution goes undetected in the average, may display more extreme photophysical characteristics [25], [36]. Long off-times of ∼10 s and more may not be fully reflected in the off-times histogram given the finite duration of the single molecule in vitro experiments and the resulting bias in favor of shorter times. Additionally, as discussed in **Text S1** and **Figure S6**, a more complex photophysical behavior could be envisioned in the presence of an additional long lived dark state. Therefore it is not possible to rule out that, at a *t_d_* value where the number of counts corresponds to the average extracted from the fit, multiple counts artifacts due to photoblinks with particularly long off-times are still present. **Figure 4** **d–g** shows how the PALM image of a small region of the cell membrane is rendered for four values of *t_d_*. At low *t_d_* molecular photoblinking is reflected in the appearance of small clusters that disappear as the allowed dark time is increases[37].

## Discussion

Starting from the complex photophysical features of one of the most recent and promising photoconvertible fluorescent proteins for PALM studies we have systematically investigated the effect of molecular photoblinking and fluorescence dark times on a typical PALM experiment. The multiple counting of even a small number of molecules may clearly have an impact in the imaging of biological systems such as proteins expressed on the plasma membrane, where phenomena such as oligomerization and clustering can be properly identified only if the same molecule is not counted multiple times.

In this work we have proposed a method to obtain a reliable estimation of the number of photoblinking molecules present in a sample by comparing simulations tailored on single molecule photophysics and in vitro experiments. We have identified a parameter, the dark time *t_d_*, depending on which it is possible to move from an overcounting to an undercounting condition, outlining different regimes to conduct a PALM measurement. We have exploited the information present in the number of localizations at different dark times *t_d_* to propose a semi-empirical equation (1) allowing us to extract the average density of blinking fluorophores from the PALM measurement. We have then applied our method to measure the number of β2 adrenergic receptors expressed on the plasma membrane of transiently transfected HeLa cells, additionally showing how images free from cluster artifacts can be obtained only by moving into the missed counts regime.

Our approach illustrates how the detailed knowledge of the fluorophore photophysical behavior can lead to a better quantification of protein numbers at the single cell level. The method has the potential to be extended to high labeling and/or activation scenarios, and eventually to other techniques such as STORM, provided that a more comprehensive treatment of the missed counts is developed, as discussed in **Text S1**. Our approach proves effective in a concentration range spanning from 10–1000 molecules/µm^2^, particularly suited for the study of plasma membrane bound receptors near their physiological concentration or above.

## Supporting Information

Text S1 Supporting information.
(DOC)

Figure S1 **Single molecule kymographs.** Typical single molecule kymographs of a mEos2 molecule in the red photo converted form under low 405 nm CW irradiation, displaying photoblinking after a long lived dark state. Molecule embedded in PAGE gel, 19∶1, pH 7.4. The two traces reflect two extreme behaviors that can be observed in the fluorescence dynamics of mEos2 single molecules, giving rise to the observed exponential distribution of on and off-times. In Figure S1 a long on and off times are observed. In figure S1 b shorter on times are separated by varying dark periods, spanning from a few hundred milliseconds to many seconds.
(EPS)

Figure S2 **On-times, off-times and number of photoblinks histograms.** **a)** Histogram of the number of times an mEos2 molecule undergoes photo-blinking before definitive photobleaching. Experimental values and single exponential best fits are shown for two different conditions of activating power (low 405 nm laser CW power and high CW power). 1/e decay values are *nblink*_low_ = 2.1±0.7, *nblink*_high_ = 1.3±0.4. Molecules are excited by a CW 561 nm diode laser in Total Internal Reflection. Superimposed markers show the result s for the blinking times produced by the simulation matching the measured histograms **b)** Histogram of measured on-times t_on-low_ = 0.138±0.007 s, t_on-high_ = 0.168±0.008 s **c)** Histogram of the measured off-times t_off-low_ = 0.37±0.05 s, t_off-high_ = 0.13±0.02 s
(EPS)

Figure S3 **Simulated PALM localization density curves as a function of post processing memory t_d_. a)** for high 405 nm photoactivation light. The effect of blinking results in a twofold reduction of the measured density as *t_d_* is increased ten times. At this density and high photoactivation rate the experiment falls in a regime dominated by missed counts. Superimposed to the simulated density curves are the experimental points corresponding to the high and low density mEos2 PAGE samples. The density ratio (blue/red) slightly above 100 times is probably due to the variability depending on sample preparation**. b)** Same as in a) but for low 405 nm photoactivation light.
(EPS)

Figure S4 **Comparison between experimental and simulated counts for varying sample density.** Experimental (markers) and simulated (blue solid line) total mEos2 molecules localized as a function of *t_d_,* time allowed for a molecule to go dark being identified as the same when fluorescence resumes. Simulated counts, out of total, ascribed respectively to missed counts (green dotted), multiple counts (pink dashed) and noise (gray, dash-dot). For all samples the duration of the acquisition is 20000×50 ms frames. **a)** concentrated sample imaged upon high power CW 405 nm activation laser light **b)** low concentration sample imaged upon high power CW 405 nm laser. In a) and b) the red curve shows best fit to data for *t_d_* values comprised between 0.05 s and 1 s. Fitting yields values for *t_off_* and N consistent with what is reported respectively in panel **c)** and **d)** extracting the off-times histograms from the counts curve. The values are in excellent agreement with what reported in Figure S2 panel b).
(EPS)

Figure S5 **Comparison between experimental and simulated counts for a dense sample.** Concentrated sample imaged under low power photoactivation light. Experimental (markers) and simulated (blue solid line) total mEos2 molecules localized as a function of t_d_, time allowed for a molecule to go dark being identified as the same when fluorescence resumes. Simulated counts, out of total, ascribed respectively to missed counts (green dotted), multiple counts (pink dashed) and noise (gray, dash-dot). The duration of the acquisition is 500000×50 ms frames. The simulated sample density is 1500 molecules/µm^2^, the concentration measured from absorbance is 25 µM. The fit yields N = 1510±80, n_blink_ = 0.67±0.08 and t_off_ = 0.22±0.05 s.
(EPS)

Figure S6 **Comparison between one vs two dark states models.** **a)** Counts vs t_d_ curve for a diluted mEos2 in PAGE sample (1 nM) displaying a single exponential decay. **b)** Markers: experimental off-times measured from single molecule traces of mEos2 in PAGE. Red dashed curve: simulated off-times for a one dark state model. Purple dotted curve: simulated off-times for a two dark states model at high photoactivation values. Green dotted curve: simulated curve for a two dark states model at low photoactivation values. **c)** Simulated counts vs td curve and for a one dark state model (blue curve) compared to a two dark states model (red curve) **d)** simulated cumulative off-time probability respectively for one (red) and two (green) dark states model.
(EPS)
