# Supplementary material for: Quantitative Photo Activated Localization Microscopy: Unraveling the Effects of Photoblinking
Source: PLoS One. 2011 Jul 26;6(7):e22678. doi: 10.1371/journal.pone.0022678 (PMC3144238; doi:10.1371/journal.pone.0022678)
Supplement: Text S1 — Supporting information. (DOC) [file pone.0022678.s001.doc]

# SuppORTING Information

## Materials and methods

**Plasmids**

The mEos2 and the PA-GFP genes are cloned into the plasmid pRSET A and were purchased from AddGene (Addgene plasmids 20341 and 11911, respectively). The Dronpa construct (in the plasmid pDG1-MN1 from MBL Medical and Biological Laboratories CO, LTD, Japan) was subcloned in the plasmid pRSET A after PCR amplification using the primers TGGGGATCCATGGTGAGTGTGATT (sense) and CTCGAATTCTTACTTGGCCTGCCT (antisense) in order to obtain the BamH I and Ecor I restriction sites.

These three constructs are cloned into an expression cassette downstream of a T7 promoter and a 6XHis region. All pRSET A plasmids were transformed into the Bacterial strain BL21(DE3) pLysS (Stratagene, La Jolla, CA, USA) for high protein expression.

For β2 receptor chimera, we fused at the C-terminus the two different fluorophores tdEos and mEos2 (β2-tdEos and β2-mEos2). For β2-tdEos, β2 sequence was amplified with PCR using the primers GATGAATTCCTTGGTACCACCATG (sense) and GGCGCGGCCGCTT CAGCAGTGAGTCATT (antisense) in order to obtain the Ecor I and Not I restrictions sites, and the deletion of the Stop codon. Then, the amplified β2 construct was inserted in the plasmid pcDNA3-td-Eos (from MoBiTec, 37083 Göttingen, Germany). For β2-mEos2, mEos2 substitutes tdEos in the β2-tdEos DNA construct.

The mEos2 gene was amplified with PCR using the primers GTCGCGCTCGAGATGAGTGCGATTAAGCCA (sense) and CGTCGCGGGCCCTTATCGTCTGGCATTGTC (antisense) in order to create the Xho I and Apa I restrictions sites. Then, we decided to use mEos2 fused to β2 because it is a monomer and brighter than tdEOS.

**Protein purification**

All pRSETa plasmids were transformed into the Bacterial strain BL21(DE3) pLysS (Stratagene, La Jolla, CA, USA). Cultures were grown overnight in Medium containing 100μg/mL ampicillin and 25μg/mL chloramphenicol to a 0.4-0.5 optical density and then induced with 1mM IPTG at 37 °C for 5 hours.

The cells were harvested from a 500 ml culture by centrifugation and resuspended in 8 ml of lysis buffer (500 mM NaCl, 50 mM NaH_2_PO_4_, pH 8). Then, 8 mg of lysozyme was added and the reaction was incubated 30 minutes on ice. The solution was sonicated on ice 5 times with a sonicator equipped with a microtip using six 10-second bursts at high intensity with a 10-second cooling period between each burst. The lysate was centrifuged at 3000 x g for 15 minutes to pellet cellular debris. The supernatant was transferred to a fresh tube and added to an equilibrated Invitrogen Ni-NTA purification Column. Binding was allowed for 1 hour with gentle agitation to keep the resin suspended in the lysate solution. The resin was allowed to settle by centrifugation (800 x g) and the supernatant was carefully aspirated. The column was washed with 8 ml of Native Wash Buffer with imidazole at low concentration (500 mM NaCl, 50 mM NaH2PO4, pH 8 and 20 mM imidazole). This last step was repeated three more times.

The column was clamped in a vertical position and the cap was removed from the lower end in order to elute the protein with 12 ml native elution buffer (500 mM NaCl, 50 mM NaH_2_PO_4_, pH 8 and 250 mM imidazole). After purification, the proteins were dialyzed in Slide-A-Lyzer cassettes with 10000 MWCO (Pierce Chemical Company, Rockford, IL) using PBS and then concentrated.

**In-vitro sample preparation**

Single molecule fluorescent proteins samples were obtained by embedding the fluorophores in a Poly Acrylamide Gel (PAGE) matrix. PAGE was obtained by diluting in water a stock solution 30% Acrylamide (Sigma) with 1.6% Methylenebisacrylamide (Sigma) to a final Acrylamide concentration of 15%. Polymerization was obtained, after a few cycles of degassing and nitrogen flow, by adding Ammonium Persulfate Solution and TEMED and mixing^1^. 20 µL of the polymerizing solution were immediately sandwiched between two 25.4 mm diameter, no. 1.5, clean glass coverslips. Fluorescence imaging was performed after 1h, and within at most a few hours, at room temperature.

**Cell cultures, transfection and sample fixation**

HeLa cells were grown in DMEM (without phenol red) supplemented with 10% fetal bovine serum, 100 µg/ml streptomycin, and 100 units/ml penicillin at 37 °C with 5% CO_2_. The cells were transfected using the NeonTM Transfection System (Invitrogen). The electroporation was performed for a 6 well dish (500,000-1,000,000 cells/well) using 0.5-2 µg of DNA for each sample following the manufacturer’s parameters (Voltage 1,005; 2 Pulses with a width of 35ms). Experiments were performed after 24 h of transfection. The day of the experiment, after extensive washing with PBS, cells were fixed incubating with 4% paraformaldehyde and 0.2% glutaraldehyde at 37°C for 30min in PHEM (60mM Pipes, 25mM HEPES, 10mMEGTA, and 2mM MgCl2, pH 6.9) and washed 5X with PHEM. The presence of glutaraldehyde was necessary to reduce molecules mobility as reported by Tanata *et al.* ^2^. The glass coverslips were washed following the protocol of Shroff et al. ^3^. The coverslips, 25.4 mm diameter, no. 1.5 thickness, were cleaned overnight in a solution obtained by mixing 125 ml of water with 25 ml of ammonium hydroxide and 25 ml of H_2_O_2_ 30%. A further step based on repeated rinsing with MilliQ water, immersion in spectroscopic grade methanol, nitrogen blowing and a passage under the flame was performed.

**Experimental Setup**

The excitation setup, built on an independent optical platform, consists of three laser-diode lines, two for excitation (488nm Coherent Sapphire 488-50 ad 561nm 50 mW Spectra-Physics Excelsior ) and one for photo-activation (405nm Coherent Cube). The excitation light is amplitude modulated and gated through an Acousto-Optical Polychromatic Tunable Filter AOM (A.A. Optoelectronics), which allows an on-off switching of each line with a rise time of 1.5 ms. Both the excitation and the activation beams are expanded (15-20X) and the activation beam is spatial filtered (pinhole size 10 µm). The filtered and expanded activation beam is combined to the excitation beam-path by a Semrock R405-Di01 dichroic Mirror. The combined laser beams are focused to the back focal plane of a 1.45 NA 100X Olympus Total Internal Reflection Fluorescence objective by a 250 mm focal length, 50 mm diameter, achromatic doublet lens placed on a x-y-z micrometric translation stage. Total internal reflection at the sample arises as the focused laser beam is translated away from the optical axis in the back focal plane of the objective, resulting in a variable exit angle of the beam with respect to the optical axis. In our setup the beam angle is accurately controlled by the rotation a 9.5 mm thick Fused Silica Laser Grade window (SQW-2037-UV Melles Griot) placed on a rotating goniometer. The goniometer is mounted in between the achromatic doublet and the back focal plane of the objective.

The microscope is based on the structure of an inverted Olympus model IX 71, with a camera side-port and an excitation port at the rear of the frame. Excitation and fluorescent wavelengths are separated by a Semrock FFT506Di02 dichroic mirror (Dronpa,PA-GFP) or a Chroma T585LP (mEos2). A Chroma ET 525/50 (Dronpa,PA-GFP) or a Semrock FF01-617/73 (mEos2) are used as emission filters.

Single molecule fluorescence images are detected by an Andor Technology iXon+ DU-897E electron multiplying CCD camera, after a 2X (or 1.2X) further magnification, yielding a pixel size of 80 nm (or 133 nm). Custom developed LabView software controls the PALM acquisition sequence, triggering the CCD detector at each excitation cycle. The CCD detector, in turn, has the possibility to trigger the AOM, to avoid any stray light on the CCD chip and therefore bleed-through during readout. Data is spooled to hard drive for post-processing.

**Imaging**

561 nm excitation power, as measured in epifluorescence mode after the objective, resulted in power densities at the sample in the range of 250-1000 W/cm^2^. Datasets were recorded by reaching to both ends of this power range. For both mEos2 and PA-GFP and Dronpa we used activation powers at 405 nm in the in the range of 10 mW (low photoactivation) to 100 mW (high photoactivation).

**Image Analysis**

Single-molecules were localized using MATLAB (MathWorks) code kindly provided by Dr. Eric Betzig and the resulting spatial point patterns were analyzed with IgorPro (Wavemetrix) custom routines. Fluorescence bursts falling within ±1 CCD pixel in space and within t_d_ frames in time were attributed to the same molecule, and collated together to prior to the molecular fitting.

**Simulation Code**

All the computational work described in the text was performed using a custom written FORTRAN software (qPALM), while fits of histograms to exponential curves where done using the suite GRACE.

To create images that simulate the ones obtained with PALM microscopy using a photoactivatable fluorophore, we used the following procedure.

First, N points representing the individual fluorescent proteins were randomly distributed in a given area representing the focal plane (35000 x 35000 nm^2^). A discrete time dynamics of the emitting state of the fluorophores, while keeping their position fixed in order to reproduce the experiments in PAGE gel or fixed cells, was then started with all fluorophores in the off state. At each time step, set to be equal to the acquisition frame-rate, each fluorophore could either remain in the off state or change to the on state in a stochastic way, such as to reproduce the activation probability arising from irradiation with 405 nm light of fixed intensity. Once activated, at each time step the fluorophores were then subjected to another stochastic process to determine whether to remain in the on state or to switch either to a reversible off state or to photobleach in a definitive way. In a similar manner, all fluorophores in the reversible off state were tested at each time step whether to remain in this off state or to turn back on. The simulation parameters for the discrete on/off dynamics were tuned such as to reproduce the following measured photophysical parameters: number of fluorophore activations per frame, on times distribution, off times distribution and distribution of number of blinks. An additive noise floor, due to false-positive localizations was also included, tailored on control experiments where the fluorophore was absent.

The on/off dynamics was then simulated for a fixed number of frames, that was set to be equal to the one used in the corresponding PALM experiment, and it was post-processed as to reproduce the observations of a PALM experiment using the following constraints: (i) a finite resolution of ±1 pixel (133 nm pixel size) around the fluorophore coordinates, such as that two simultaneous emitting fluorophores within this distance were detected as a single fluorophore, (ii) an allowed off time (postprocessing memory t_d_) such that consecutive reactivations of the same fluorophore (or of a different fluorophore within the same pixel) within the allowed off time were considered as a single detection instead of two consecutive ones (a multiple count for the same fluorophore or a missed count in the case of two colocalized molecules), (iii) a detection error obeying to a uniform distribution within ± [0;20] nm in x and y coordinates.

Finally, the comparison between the initial randomly generated spatial distribution of fluorophores and the output from the simulated PALM experiment was used to detect the effects induced by fluorophore photoblinking, namely, the number of multiple counts and the number of missed counts.

**Photophysical model**

In the current work the photophysical scheme for the fluorescent protein of our choice is that of a four state system, namely inactivated, activated, dark, and bleached. Inactivated to activated is an irreversible photoconversion process, and once the molecule is photoactivated the inactive state does not play any further role in the fluorescent dynamics of the protein. Once photoconverted a fluorophore such as mEos2 displays a bright red fluorescence, which can disappear upon the entrance of the molecule in a dark state, and fluorescence recovery appears to be favored by 405 nm irradiation. The off-times histograms appear compatible with a single rate, consistent with a four state hypothesis. It shall be noted that in any widefield experiment the detector integration time, typically in the range of tens of milliseconds, (50 ms in our case) will filter out all faster dynamics. This will in general apply to triplet blinking that reportedly falls on a timescale one order of magnitude shorter than this value.

The four state model represents a first-order variation from the ideal photoactivatable fluorophore model envisioned in designing the PALM concept, namely a protein that is dark until photoactivated, emits continuously and then irreversibly photobleaches.

Although in the current work the four state model appears to explain most of the observations, it is possible that a more complex dynamics is present, or that the four states assumption will result inadequate in the presence of a more complex photophysical behavior. This would be the case for a reversible photoswitching molecule, where two dark states are present e.g. a photoswitched-off state and an off state of shorter duration from which the protein can spontaneously recover. This is, for example, the case of Dronpa, displaying reversible 405 induced photoswitching and an additional fast-blinking with an average off-time value of tens of milliseconds ^4^.

Our fitting method will take into account the reactivations resulting from the faster of the two dynamics, but in the current form will not consider the slower one. The weight of the slower dynamics and the validity of a four states model can be assessed before hand by observing the distribution of the off-times of the molecule.

SI Figure 6 a displays the counts vs t_d_ curve (introduced in Figure 1 a) for a very diluted mEos2 sample in PAGE (1nM). This condition rules out the presence of a significant fraction of missed counts. Counts vs t_d_ behavior is observed up to t_d_=500 s, and results in a monoexponential decay. Figure S6 b displays the experimentally measured off-time histogram compared to simulations of both a four state model, as the one used in our work, and a model with an additional dark state (for both a low and a high photoactivation value). The experimental points are in good agreement with either a four state model or a low photoactivation two dark states model. Figure S6 c displays the comparison between the simulated counts vs t_d_ curves of a two dark states model and the four state model expected for the same photoactivation value. The resulting curves display that the choice of the model is not dramatically influencing the shape of the curves.

The amount of re-activations events due to the slower dynamics can in this case be estimated from the cumulative probability extracted from the off times histogram. Figure S6 d displays how, assuming a two dark states model compatible with our data would result only in approximately 10% of the events displaying off-times longer than 2 s, the cut-off value observed in the three-state simulation.

**Semi Empirical fitting function**

As Figure S2 displays, the off-times histogram can be reconstructed from the PALM counts vs. dark time *t_d_* curve. As a preliminary step we call c(*t_d_*) the number of localized molecules at a given dark time *t_d_* and for simplicity we construct the off-times histogram using a bin-size corresponding to dark time *t_d_.* Here we will refer to the *t_d_* and *t_off_* as of multiples of the frame rate acquisition time (i.e. *t_d_*=1 is equivalent to *t_d_* =50 ms in the text, *t_d_* =2 is equivalent to *t_d_* =100 ms and so on...).

It is then straightforward to recognize that $c(t_{d}=1)-c(t_{d}=2)=A\cdot\exp(-\frac{t_{d}=2}{t_{off}})$, since the difference in counts extending of one integration time dark time t_d_ yields the number of off-times of 1 CCD frame extracted from single molecule traces.

We call N the total number of molecules effectively present in the sample, and n_blinks_ the average number of re-activations undergone by a given molecule (here blinks and reactivations are used with the same meaning).

We have: $c(t_{d}=n)-c(t_{d}=n+1)=A\cdot\exp(-\frac{t_{d}=n+1}{t_{off}})$ if we treat the above as a continuous expression (in the limit of dark time t_d_ going to 0) we can integrate it obtaining: $c(t_{d})=A\cdot t_{off}\cdot\exp(-\frac{t_{d}=n+1}{t_{off}})+B\cdot t_{off}$using as a boundary conditions: $c(t_{d}=1)=N+N\cdot n_{blink}$and $c(t_{d}=\infty)=N$ we obtain: $N\cdot(1+n_{blinks})=A\cdot t_{off}\cdot\exp(-\frac{t_{d}}{t_{off}})+B\cdot t_{off}$ where $N=B\cdot t_{off}$and therefore we recover the proposed equation (1) in the main text: $c(t_{d})=N+N\cdot n_{blinks}\cdot e^{\left(\frac{1-t_{d}}{t_{off}}\right)}$

It has to be noted that the boundary condition $c(t_{d}=\infty)=N$assumes that no missed counts occur. Therefore it is important, as pointed out in the text, to perform the fit to equation in the overcounting regime.

In the presence of a dense sample, there will be a photoactivation value above which a non-negligible fraction of missed counts may occur. We propose here a simple consideration to address this case. Calling the temporal activation density *n* (number of activated molecules every *x* frames in the diffraction limited region) and assuming that the activation process follow a Poisson process, the probability of two different molecules falling within the same interval t_d_ is proportional to (1-exp(-n*t_d_)). For t_d_ going to infinity the number of missed molecules should account for most of the molecules in the sample aside from the very first localizations occurred in each DLR.

It could be possible to extend equation (1) to take into account missed counts in the way that:

$$N(t_{d})=N\cdot(1+n_{blink}\cdot e^{\left(\frac{1-t_{d}}{t_{off}}\right)})-N©\cdot(1-e^{-n\cdot(t_{d}-1)})$$

However this would require an increased number of parameters to be taken into account and the inclusion of additional parameters could lead to overfitting problems.

**References**

1 Chrambac.A & Rodbard, D. Polyacrylamide Gel Electrophoresis. *Science* **172**, 440-& (1971).

2 Tanaka, K. A. K. *et al.* Membrane molecules mobile even after chemical fixation. *Nat Methods* **7**, 865-866, doi:Doi 10.1038/Nmeth.F.314 (2010).

3 Shroff, H., White, H. & Betzig, E. Photoactivated localization microscopy (PALM) of adhesion complexes. *Curr Protoc Cell Biol* **Chapter 4**, Unit 4 21, doi:10.1002/0471143030.cb0421s41 (2008).

4 Habuchi, S. *et al.* Reversible single-molecule photoswitching in the GFP-like fluorescent protein Dronpa. *P Natl Acad Sci USA* **102**, 9511-9516, doi:Doi 10.1073/Pnas.0500489102 (2005).
